# MR Volumetric Study of Piriform-Cortical Amygdala and Orbitofrontal Cortices: The Aging Effect

**DOI:** 10.1371/journal.pone.0074526

**Published:** 2013-09-12

**Authors:** Jing Shen, Mohammad A. Kassir, Jianlin Wu, Qing Zhang, Shiyu Zhou, Stephanie Y. Xuan, Qinghang Li, Yongquan Ye, Jiani Hu

**Affiliations:** 1 Department of Radiology, Affiliated Zhongshan Hospital of Dalian University, Dalian, China; 2 Department of Radiology, Wayne State University, Detroit, Michigan, United States of America; 3 Department of Mental Health, Dalian medical University, Dalian, China; 4 University of Toronto, Faculty of Arts & Science, Toronto, Ontario, Canada; 5 Department of Neurological Surgery, Wayne State University, Detroit, Michigan, United States of America; Cardiff University, United Kingdom

## Abstract

**Introduction:**

The piriform cortex and cortical amygdala (PCA) and the orbitofrontal cortex (OFC) are considered olfactory-related brain regions. This study aims to elucidate the normal volumes of PCA and OFC of each age groups (20.0-70.0 year old), and whether the volumes of PCA and OFC decline with increasing age and diminishing olfactory function.

**Methods:**

One hundred and eleven healthy right-handed participants (54 males, 57 females), age 20.0 to 70.0 years were recruited to join this study after excluding all the major causes of olfactory dysfunction. Volumetric measurements of PCA and OFC were performed using consecutive 1-mm thick coronal slices of high-resolution 3-D MRIs. A validated olfactory function test (Sniffin’ Sticks) assessed olfactory function, which measured odor threshold (THD), odor discrimination (DIS), and odor identification (ID) as well as their sum score (TDI).

**Results:**

The volume of OFC decreased with age and significantly correlated with age-related declines in olfactory function. The volume of OFC showed significant age-group differences, particularly after 40 years old (p < 0.001), while olfactory function decreased significantly after 60 years old (p < 0.001). Similar age-related volumetric changes were not found for PCA (p = 0.772). Additionally, there was significant correlation between OFC and DIS on the Right Side (p = 0.028) and between OFC and TDI on both sides (p < 0.05). There was no similar correlation for PCA.

**Conclusions:**

Aging can have a great impact on the volume of OFC and olfactory function while it has much smaller effect on the volume of PCA. The result could be useful to establish normal volumes of PCA and OFC of each age group to assess neurological disorders that affect olfactory function.

## Introduction

The piriform cortex and cortical amygdala (PCA) and the orbitofrontal cortex (OFC) are two important olfactory-related brain regions. The PCA is considered a primary olfactory cortical region as it receives direct input from the olfactory bulb [1]. The OFC receives odor input from the piriform cortex and forms reciprocal connections allowing it to modulate piriform cortical activity [2,3]. These brain regions form a network that responds to odors based on odor memory and motivational state. Most recent research on odor-evoked cortical activity has focused on these two areas of the brain [1].

Olfactory function has been found to have a tendency to diminish with advancing age [4,5]. It has also been observed to be affected significantly by multiple disease processes [5]. Concurrent use of olfactory function tests and volumetric MRI studies can assess the relationship between changes in olfactory function and changes in olfactory-related brain regions. For example, it has been revealed that olfactory bulb volume significantly decreases with age and that the correlation between olfactory function and olfactory bulb volumes is independent of age [6]. However, few studies have been done to examine whether the volumes of PCA and OFC decrease with age, and decline in parallel to olfactory function [7,8].

The present study has accomplished two objectives by combining manual measurements of PCA and OFC volume using MRI with the 'Sniffin' Sticks’ test for olfactory function [9-11]. The first objective is to establish normative volume values of PCA and OFC for different age groups and assess corresponding olfactory function. The second goal is to investigate any possible relationships between changes in the volume of PCA or OFC and changes in olfactory function with increasing age.

## Methods

### Ethics Statement

The study was approved by the Committee on Medical Ethnics of the Affiliated Zhongshan Hospital of Dalian University, and performed in accordance with the ethical guidelines of the Declaration of Helsinki. After complete description of the study to the subjects, written informed consent was obtained from each subject.

### Subjects

One hundred and eleven healthy right-handed participants (54 males, 57 females) ages 20.0 to 70.0 years (mean: 44.2 ± 14.0 years) were enrolled in the study. All the subjects were carefully screened for major causes of olfactory dysfunction using otorhinolaryngological examinations including nasal endoscopy and a questionnaire. None of the participants had a history of head injury, hypertension, neurodegenerative disease, diabetes mellitus, or recent upper respiratory tract infection. Initially, a total of 144 volunteers were recruited. Reasons for rejection are as follows: rhinosinusitis identified on MRI examination (9 persons), neurodegenerative diseases (11 persons), recent upper respiratory tract infection (3 persons), history of head injury (1 person), left-handedness (2 persons), and cerebrovascular disease (7 persons).

The remaining 111 subjects were divided into five age groups: group A (20.0-29.9 years, mean age: 25.3 ± 2.4 years; 10 males and 12 females), group B (30.0-39.9 years, mean age: 34.2 ± 2.7 years; 14 males and 10 females), group C (40.0-49.9 years, mean age: 44.7 ± 3.2 years; 10 males and 10 females), group D (50.0-59.9 years, mean age: 54.2 ± 3.0 years; 10 males and 16 females), and group E (≥ 60.0 years, mean age: 64.4 ± 2.7 years; 10 males and 9 females). The criteria for normative olfactory function were according to the research of Dr. Hummel [11]; the TDI score was 24.9 for age < 15years, 30.3 for age 16-35years, 27.3 for age 36-55years, and 19.6 for age > 55years.

### MRI Data Acquisition

The MRI scans were acquired on a 1.5T GE HD Signa MR scanner. A three-dimensional fast spoiled gradient-recalled sequence (3D-FSPGR) was used for acquisition of 160-190 contiguous T1-wighted slices of 1.0mm thickness in the sagittal plane. The imaging parameters were as follows: repetition time (TR) = 9.6 ms; echo time (TE) = 4.2 ms; flip angle = 15°; number of excitations (NEX) = 1; field of view (FOV) = 256 mm; slice thickness = 1mm; slice interval = 0 mm; matrix size = 256 × 256. The voxel size was 1.0 × 1.0 × 1.0mm.

All raw data were transferred to a Unix workstation (Silicon Graphics, Mountain View, CA, USA) and processed with the software package Dr View 5.0 (Asahi Kasei Joho System, Tokyo, Japan). Brain images were realigned in three dimensions and reconstructed into entire contiguous coronal slices of 1mm thickness (voxel size = 1.0 × 1.0 × 1.0 mm) perpendicular to the brainstem and cerebellum. The signal intensity histogram distributions across the whole cerebrum segmented the voxels semi-automatically into gray matter, white matter, and cerebrospinal fluid (CSF).

### Volumetric Analysis of Regions of Interest

**Figure 1:**
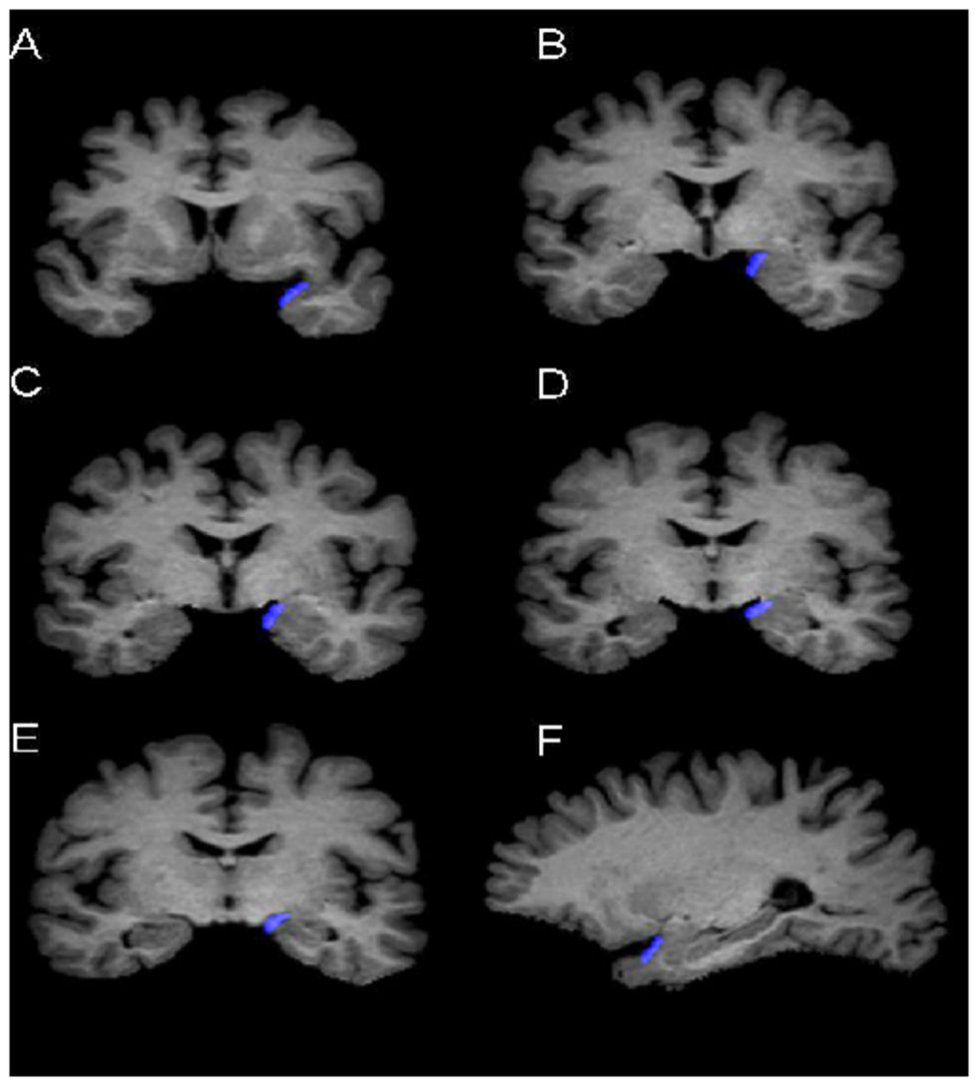
Delineation of the piriform-cortical amygdala (PCA). Sample views of the PCA (in blue) at different coronal levels (A-E) and a sagittal level (F). Sagittal views were automatically reconstructed synchronously when the delineation was performed on consecutive coronal slices.

**Figure 2:**
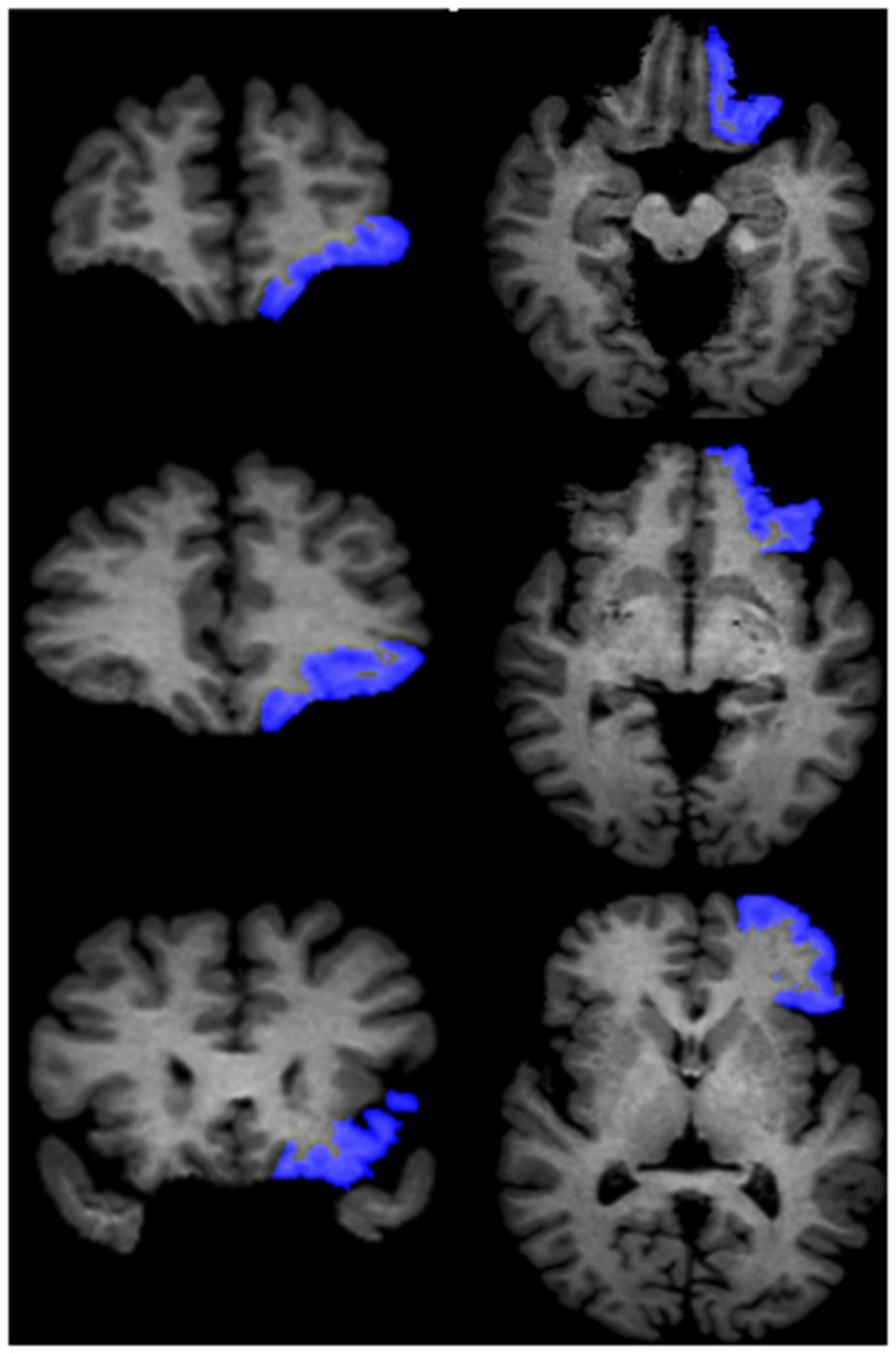
Delineation of the orbitofrontal cortex (OFC). Sample views of the OFC (in blue) at different coronal levels (left column) and transaxial levels (right column). Transaxial views were automatically reconstructed synchronously when the delineation was performed on consecutive coronal slices.

Separate manual volumetric measurements were performed on the PCA and OFC in the left and right hemispheres using consecutive 1-mm-thick coronal slices, with the corresponding sagittal and axial planes presented simultaneously for assurance of landmarks and integrity of the ROIs as defined below (Figure 1 and Figure 2).

#### Piriform cortex and cortical amygdala (PCA)

The initial portion of the Piriform Cortex (PC) is located in the caudolateral portion of the orbitofrontal cortex, and extends to the dorsal medial temporal lobe. The frontal portion of the PC occupies only a small portion of total PC volume (10-15%) [12], and there are no clues to outline the borders through MRI. Therefore, the definition for PC in the present study comprised only its temporal portion, and PC and cortical amygdale (PCA) were considered as one region of interest (ROI) in our study. This is method was based on that used by Goncalves Pereira et al. for delineation of PCA [13].

The PCA was outlined in the rostral to caudal direction. The appearance of the limen insulae (LI, or the junction between the frontal and temporal lobes) marked the most anterior slice of the PCA. The most posterior slice was at the level of the opening of the hippocampal fissure (HF) in the coronal section. The lateral border of the PCA was the fundus of the entorhinal sulcus, and the medial border was at the most convex point of the medial temporal cortex just before the appearance of amygdala. When the amygdala became visible, we took the most medial point of the gyrus semilunaris as the medial border. If the sulcus semiannularis was clearly present, the fundus of the sulcus was considered as the medial border [13]. In the cases were the sulcus semiannularis was not visible, the approximate medial border was determined by the intersection of the line extending from the white matter under the amygdala to the surface of the brain [13,14]. The thickness of PCA was determined by the increase in tissue signal (before the amygdala appeared) or where the contour of the amygdala was clearly present.

#### Orbitofrontal Cortex (OFC)

One author of this study (Zhou) successfully replicated Crespo-Facorro’s et al. method for delineation of the OFC [15] using the software Dr. View in 2005. The medial boundary was taken as the deepest point of the olfactory sulcus (OS) on the intermediate and posterior portions of OFC. On the anterior portion, when the OS is no longer visible, the deepest point of the superior rostral sulcus (SRS) represented the medial boundary of the OFC. For the lateral boundary, on the anterior portion of OFC, the frontomarginal sulcus (FMS) was considered as the lateral boundary before the appearance of the lateral orbital sulcus (LOS). When the LOS was clearly seen in the intermediate aspect of the frontal lobe, the deepest point of LOS constituted the lateral boundary of the OFC. When the LOS is no longer visible on coronal slices, the inferior margin of the circular sulcus of the insula (CSI) was defined as the lateral boundary of the OFC on its caudal portion. Tracing ended at the most posterior coronal slice containing some aspect of the posterior medial orbitofrontal gyrus that corresponded to the most posterior edge of the OFC.

Volumetric measurements were done by one trained rater (J.S.). The researchers were blinded to the subjects’ identifiable information. Reliability of the ROIs was established by intra-class correlation analysis of the two structures (PCA:0.98; OFC:0.94).

#### Olfactory Function Test (Sniffin’ Sticks)

"Sniffin' Sticks" assessed olfactory function [9-11]. This olfactory function test uses pen-like odor dispensing devices to test three aspects of olfactory function: odor threshold (THD), odor discrimination (DIS), and odor identification (ID) as well as their sum score (TDI). The test-retest reliability of this assay has been established by previous studies [9-11]. The “Connecticut Chemosensory Clinical Research Center Test” (CCCRC) [16] and the “Cross Cultural Smell Identification Test” (CC-SIT) [17] demonstrated its validity in comparison to established tests of olfactory function [9]. Many previous studies and clinicians use this test [11]. "Sniffin' Sticks" is a reliable test and the method of choice for measuring olfactory function in the present study.

For odor presentation, the cap was removed for about 3 seconds and the pen’s tip was placed about 2 cm in front of one nostril while the other nostril was covered with tape. To prevent other possible influencing factors, a sleeping mask was used for subjects during the test, and gloves were used by the investigators. Testing was always performed in quiet, well-ventilated rooms.

Odor thresholds for n-butanol were assessed using a single-staircase, three alternative forced choices (3-AFC) procedure. Sixteen dilutions were prepared in a geometric series starting from a 4% n-butanol solution (dilution ratio 1:2). Three pens were presented randomly, subjects had to correctly identify the odor-containing pen twice (two pens contained solvent and the third odorant). Correct or incorrect identification then triggered a reversal of staircase to the next higher or lower dilution, respectively. The odor threshold result was determined as the mean of the last 4 staircase reversals.

In the odor-discrimination task, 16 sets of three pens were presented in a randomized order. Subjects had to discriminate which of the three pens smelled differently (two contained the same odorant while the third one was different). The score of odor-discrimination was determined by the sum of correctly identified pens out of 16 sets.

For the odor-identification test, we used a version of odor identification test which had been modified for Asia The “Sniffin’ Sticks” test originated in Europe and because of different living habits between Europe and Asia, the set of odors was modified. The 16 odors that we adopted were those chosen by Veterans-General-Hospital (VGH) version of identification test, which had proved to be reliable and valid in assessment of odor identification in northeast Asia [18]. Each of the odors was presented with a list of four descriptors. The subjects had to identify the correct descriptor for the presented odor. The interval between odor presentations was 20-30s. Again, the subjects’ scores were the sum of correctly identified odors out of 16 sets.

Results of the three subtests were grouped together as a composite of “TDI scores”, which was the sum of the results of odor-threshold, discrimination, and identification. 

### Statistical Analysis

Statistical differences in the volumetric measurements of the PCA and OFC across age groups were analyzed by repeated multivariate analysis of covariance (MANCOVA), with intracranial volume (ICV) as a covariate, age-group (group A-E) and gender (male, female) as between-subject factors, and hemisphere (left, right) as a within-subject factor. Age-group differences in olfactory function were analyzed using repeated MANOVA, with age-group and gender as between-subject factors, and olfactory measure (left, right) as a within-subject factor. Post-hoc Tukey’s honestly significant difference test modified for unequal sample sizes was employed to follow up the significant main effects or interactions yielded by these MANCOVAs and MANOVAs.

For the correlation analysis, relationships between age and the ROI volumes were explored by two ways: (1) Applying Pearson’s partial correlation controlling for ICV, we evaluated the correlations between the absolute volumetric measures and age; (2) Using Pearson’s bivariate correlation coefficients to access the relations between the relative volumetric measures (100 × absolute volumetric value / whole cerebral gray matter) and age. Pearson’s partial correlation analyses controlling for ICV were used for the relationship between the ROIs’ volumes and olfactory function.

## Results

Volumes of PCA, OFC, scores of Sniffin’ Sticks, and results of age-group comparisons using MANCOVA or MANOVA are presented in Table 1 and Table 2, respectively.

**Table 1:** Volumes of the intracranial cavity, cerebral hemisphere, cerebral gray and white matter, piriform and cortical amygdala (PCA) and orbitofrontal cortex (OFC) in each age group.

ROI	Group A	Group B	Group C	Group D	Group E	Main effects of age group		
	(20-29.9y)	(30-39.9y)	(40-49.9y)	(50-59.9y)	(≥60y)	*F*	*df*	*p*
ICV	1481.0±141.7	1478.3±151.2	1409.4±118.5	1409.3±151.8	1420.4±123.0	1.788	4,101	0.137
WCH	1136.6±128.6	1124.8±117.3	1060.8±86.5^§^	1029.8±108.3^§^	1010.6±79.3^§^	12.130	4,100	<0.001
WCGM	716.7±102.5	707.9±84.6	655.1±66.2^§^	645.8±62.3^§^	643.8±49.9^§^	3.417	4,100	0.012
WCWM	419.9±62.3	416.9±62.1	405.7±41.2	384.0±66.0	366.8±42.8^△^	3.238	4,100	0.015
PCA						0.451	4,100	0.772
Left	0.499±0.053	0.515±0.065	0.489±0.050	0.488±0.043	0.484±0.051			
Right	0.508±0.045	0.521±0.059	0.503±0.037	0.506±0.044	0.512±0.043			
OFC						5.908	4,100	<0.001
Left	14.458±2.217	14.267±2.320	13.111±2.063^#^	12.083±1.558^#^	11.990±2.769^#^			
Right	14.102±2.150	13.823±2.204	12.720±1.983^#^	12.168±1.824^#^	11.665±2.232^#^			

Values represent mean ± SD of measured volume (cm3). Post hoc comparisons following multivariate analysis of variance with intracranial volume as covariates (MANCOVA) revealed: ^§^p<0.001, smaller than group A and B; ^△^p<0.05, smaller than group A, B, and C; ^#^p<0.01, smaller than group A and B.

ROI: region of interest; ICV: Intracranial volume; WCH: Whole cerebral hemisphere; WCGM: Whole cerebral gray matter; WCWM: Whole cerebral white matter

**Table 2:** Scores of odor threshold, odor discrimination, odor identification and the sum score.

ROI	Group A (20-29.9y)	Group B (30-39.9y)	Group C (40-49.9y)	Group D (50-59.9y)	Group E (≥60y)	Main effects of age group		
						*F*	*df*	*p*
Threshold						6.413	4,101	<0.001
Left	8.23±1.29	7.90±1.12	7.93±1.78	7.54±1.58	6.21±1.76*			
Right	8.40±1.86	8.20±0.99	8.10±1.01	7.91±2.01	6.59±1.62*			
Discrimination						5.248	4,101	<0.001
Left	9.86±2.19	9.04±1.49	8.99±1.77	9.05±2.25	7.59±2.23^△^			
Right	9.73±1.96	9.08±1.41	9.24±1.74	9.26±2.26	7.22±1.71*			
Identification						15.691	4,101	<0.001
Left	15.00±1.11	14.04±1.40	13.91±2.08	12.31±2.46^﹟^	11.18±1.96^﹟^			
Right	15.05±1.09	14.00±1.41	13.79±1.90	12.66±2.45	11.29±1.58^﹟^			
Sum scores (TDI)						20.606	4,101	<0.001
Left	33.09±2.78	30.98±2.33	30.83±3.97	28.90±3.51	24.99±4.96*			
Right	33.17±3.11	31.28±1.93	31.13±2.53	29.83±4.51	25.11±3.73*			

Values represent mean ± SD of tested scores. Post hoc comparisons following multivariate analysis of variance revealed: *p<0.01, lower than group A, B, C and D on the ipsilateral side of each olfactory function. ^△^p<0.05, lower than group A of odor discrimination on the left side; ^#^p<0.001, lower than group A and B; ^*^p<0.001, lower than group A, B, C, and D. The mean difference is significant at the 0.05 level.

### Volumes of PCA and OFC

MANCOVA with ICV as a covariate revealed no significant correlation between PCA volume and age-group (F4, 100 = 0.451, p = 0.772) or gender (F1, 100 = 0.517, p = 0.474). However, a significant effect of hemisphere was detected for PCA (F1, 101 = 11.646, p < 0.001), with left PCA being significantly smaller than the right in participants.

**Figure 3:**
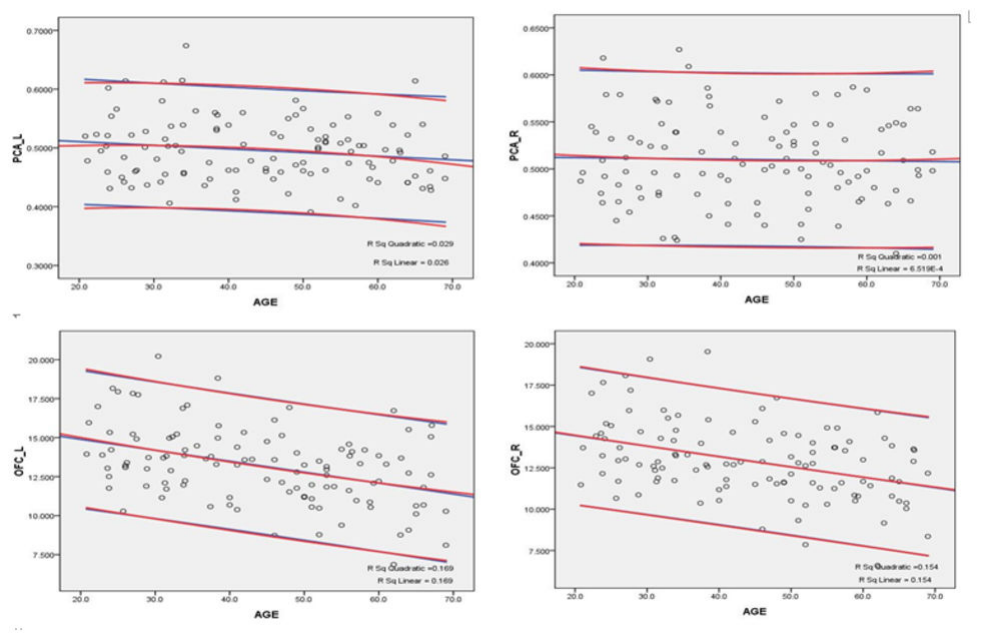
Scatter plots and regressions of values of the PCA volume and OFC volume by age with the 95% CI. PCA_L: p = 0.187; PCA_R: p = 0.631; OFC_L: p < 0.001; OFC_R: p < 0.001.

For the volume of OFC, a significant effect was observed for age (F4, 100 = 5.908, p < 0.001), as well as an effect of hemisphere (F1, 101 = 6.407, p = 0.013). Post-hoc analyses demonstrated that the volumes of OFC in the 4th (age-group C), 5th (age-group D), and 6th (age-group E) decades of age were significantly smaller than those of younger age-group participants (p < 0.001 for all between-group comparisons on both sides). And the OFC volume of the left side was larger than the right side. There was no significant effect of gender on PCA OFC volume (F1, 100 = 0.341, p = 0.561). No age-related volumetric changes were found in PCA (Figure 3).

### Correlations between PCA and OFC volume and age

Pearson’s partial correlation coefficients controlling for ICV revealed that there was no significant correlation between age and the absolute volumetric measures of PCA (left: r108 = -0.127, p = 0.187; right: r108 = 0.046, p = 0.631), but significant negative correlations were present between age and the absolute volumetric measures of OFC on both sides (left: r108 = -0.387, p < 0.001; right: r109 = -0.364, p < 0.001). Moreover, significant correlations were found between age and the relative volumetric values (100 × absolute volumetric values / whole cerebral gray matter) of right PCA (r111 = 0.325, p = 0.001) of OFC on both sides (left: r111 = -0.252, p = 0.008; right: r111 = -0.218, p = 0.022). The scatter plots and regressions of OFC and PCA volumes by age are shown in Figure 3.

### Results of Sniffin’ Sticks

Repeated MANOVAs revealed significant effects of age on all 3 types of olfactory function and their sum score: THR (F4, 101 = 6.413, p < 0.001), DIS (F4, 101 = 5.248, p < 0.001), ID (F4, 101 = 15.691, p < 0.001), and TDI (F4, 101 = 20.606, p < 0.001). Post-hoc analyses demonstrated that, except for the left ID score which became significantly lower after age of 50, the other scores including bilateral THR, bilateral DIS, right ID, and bilateral TDI became significantly lower after age of 60 compared to the younger groups.

### Correlations between PCA and OFC volume and olfactory function

Pearson’s partial correlation analyses controlling for ICV revealed a significant correlation between OFC and DIS on the right side (r_108_ = 0.209, p = 0.028), and a trend-level significant correlation on the left side (r_108_ = 0.165, p = 0.085). The correlations between OFC and TDI were significant on both sides (left: r_108_ = 0.229, p = 0.016; right: r_108_ = 0.238, p = 0.012). A trend-level significant correlation between OFC and ID was also observed bilaterally (left: r_108_ = 0.171, p = 0.075; right: r_108_ = 0.169, p = 0.077). No significant correlation was detected between the volume of PCA and olfactory function. The correlation analysis between the relative volumes and olfactory scores showed only significant relations between the right OFC and right TDI total score (r_108_ = 0.201, p = 0.034).

## Discussion

The results obtained in the present study reveal several significant findings. First, a significant negative correlation was shown between age and the volumes of OFC on both sides of the brain with OFC volumes becoming significantly reduced after 40 years old. No correlation was seen between age and volumetric measurements of PCA on either side. Second, significant negative correlations were observed between age and the scores of all three parameters of the "Sniffin' Sticks" test. Olfactory function declined significantly after the age of 60 in both nostrils. Third, there was significant correlation between OFC and DIS on the right side, and a trend-level significant correlation on the left side. In addition, the correlations between OFC and TDI were significant on both sides. No significant correlation was detected between the volume of PCA and olfactory function. To our knowledge, this is the first study to report normative data for PCA and OFC volumes for different age groups (20.0-70.0 year old), and to show significantly reduced OFC volume after 40 year old.

The PCA is thought to be a part of the primary cortex of olfaction while OFC is thought of as the central cerebral cortex [19,20]. There are many studies on the correlation between structural measurement in the brain and olfactory function in the present literature [7,21-24]. However, there is no study that takes into account the three parameters looked at in this study (volume of PCA or OFC, olfactory function, and age) all together systemically. In the present study, we evaluated PCA and OFC volumes as well as olfactory function and looked at correlations between age and ROI volumes, ROI volumes and olfactory function, and olfactory function and age.

One study that is similar to the present one is that of Seubert et al. [7]. However, in Seubert’s study, all subjects were treated as one age group (mean age: 37 ± 17.3 years old) and PCA or OFC volumes were not analyzed for changes with increasing age. There are also other differences between the two studies that pertain to the technical details. The method used for volume measurement by Seubert et al. is voxel-based morphometry (VBM) while manual volumetric MRI was used in the present study. VBM images require normalizing and smoothing, and inaccuracy in local areas and template matching can lead to systematic error and even incorrect statistical conclusions [25]. In contrast, our method is based on real anatomical boundaries, which can be easily obtained using the software of Dr. View [26-28]. Nevertheless, the two studies resulted in the same conclusion regarding the correlation between the volume of OFC and olfactory function.

The mechanism behind the observation of why OFC volume decreases from the 4th decade and olfactory function only from the 6th decade is not known. It is universally accepted that the cerebral gray matter (including cerebral cortex and most sub-cortical structures) begins to shrink after the 2nd decade of life. Also, there is evidence that functional deterioration of OFC is more vulnerable to the effects of aging than most other regions of the brain [29]. Raz et al. suggested that age-related deterioration of structures that receive dense cholinergic projections from the Nucleus Basalis of Meynet maybe the cause of age-related volume decrease [29]. On the other hand, olfactory function is considered to be associated with not only the volume of OFC, but also the volumes of the other olfactory-related regions. Similarly, the exact mechanism behind the relative stability of PCA volume with age is not known. A simple speculation is that PCA may be a region which degenerates slower or remains relatively stable with age compared to other areas of the cerebral cortex, as has been shown to be the case in the rat brain [30].

In our study, all parameters of the "Sniffin' Sticks" test showed significant negative correlations with age. The decline in olfactory function was most pronounced after 6th decade of age. This finding is in agreement with what is known about changes in olfactory function with age [4,5] This result is also comparable with Hummel’s work [11] on normative data for the "Sniffin’ Sticks" scores using a very large sample. It is also congruent with the study done by Shu et al [18].

Our data also showed significant correlations between OFC volume and both odor discrimination and overall olfactory function. The human OFC has been reported to play a guide role in olfactory exploration [31], and to be partially involved in decision making [32,33]. When the OFC is surgically removed, both monkeys and humans show strikingly decreased odor discrimination ability [34,35]. Furthermore, Tanabe et al. [36] concluded that the cells in the monkey’s lateral posterior orbital cortex were odor selective. The critical role of the PCA in olfactory information processing has also been evidenced by both animal studies and human studies [37-39]. When the temporal lobe is removed in humans, they show a significant deficit in odor discrimination [35]. However, PCA volume did not appear to vary with changes in olfactory function, as concluded by Seubert in their study [7]

The olfactory bulb and olfactory tract have been studied using similar techniques to those used in the current study. In a study by Buschhuter et al. [6] olfactory function tests and volumetric MRI were combined to study the relationship between changes in olfactory function, olfactory bulb and olfactory tract volumes, and age. Buschhuter’s study established normative data for olfactory bulb and olfactory tract volumes and found the olfactory bulb volume to significantly decrease with age. Olfactory bulb volumes were stable up to and peaked in the fourth decade of age and declined in the sixth and seventh decades. In addition, Buschhuter’s study displayed a correlation between olfactory function and olfactory bulb volume that is independent of age. The present study found similar trends between OFC volume and age.

Olfaction has been shown to be affected in many disorders. Patients with Alzheimer’s disease have decreased olfaction. In fact, the olfactory system seems to be one of the most affected by deposition of neurofibrillary tangles and neuritic plaques. A decreased ability to identify and detect odors presents early in the course of Alzheimer’s disease and worsens with disease progression [5]. Also, Parkinson’s disease (PD) patients have been found to have alterations in smell function [5]. Olfactory dysfunction has been recognized as one of the earliest non-motor features of PD [40]. One recent study found severe hyposmia as a risk factor for development of dementia within 3 years in PD patients [41]. In the same study, MR volumetric studies showed close relationships between olfactory dysfunction and the atrophy of focal brain structures including the amygdala [41]. In addition, hyposmia has been found to be a common feature of spino-cerebellar ataxias [42]. Furthermore, acute and severe depression may be associated with a decrease in olfactory sensitivity as well as a reduction in olfactory bulb volume [43]. Other volumetric studies have shown olfactory bulb volumes to be decreased in patients with post-infectious olfactory dysfunction, post-traumatic olfactory dysfunction, and schizophrenia [44-46]. The data obtained from the present study and other similar studies could be useful in detecting such disease processes in their early stages.

This research has limitations. First, the research was conducted only on a small size of population, particularly in terms of each age-group. Second, we did not use Bonferroni correction for multiple comparisons, because whether the P value needed to be adjusted for the multiple comparisons remains controversial [47]. Some researchers insist the necessarily of the Bonferroni correction for multiple comparison [47,48]. But others believe it is overly conservative [47,49,50], and statistical results based on not only the statistical significance (adjusted or not), but also upon the quality of the research within the study [47,50]. In this study the P value was set the same as Buschhuter’s correlation study between olfactory bulb volume and olfactory function [6].
